# Dietary Sugars Analysis: Quantification of Fructooligossacharides during Fermentation by HPLC-RI Method

**DOI:** 10.3389/fnut.2014.00011

**Published:** 2014-07-31

**Authors:** Daniela M. Correia, Luís G. Dias, Ana C. A. Veloso, Teresa Dias, Isabel Rocha, Lígia R. Rodrigues, António M. Peres

**Affiliations:** ^1^Centre of Biological Engineering (CEB), University of Minho, Braga, Portugal; ^2^Centro de Investigação de Montanha (CIMO), Escola Superior Agrária, Instituto Politécnico de Bragança, Bragança, Portugal; ^3^Departamento de Engenharia Química e Biológica (DEQB), Instituto Superior de Engenharia de Coimbra, Instituto Politécnico de Coimbra, Coimbra, Portugal; ^4^Laboratory of Separation and Reaction Engineering (LSRE) – Associate Laboratory LSRE/LCM, Escola Superior Agrária, Instituto Politécnico de Bragança, Bragança, Portugal

**Keywords:** dietary sugars, fructooligossacharides, HPLC-RI method, yield fermentation process, in-house validation

## Abstract

In this work, a simple chromatographic method is proposed and in-house validated for the quantification of total and individual fructooligossacharides (e.g., 1-kestose, nystose, and 1^F^-fructofuranosylnystose). It was shown that a high-performance liquid chromatography with refractive index detector could be used to monitor the dynamic of fructooligossacharides production via sucrose fermentation using *Aspergillus aculeatus*. This analytical technique may be easily implemented at laboratorial or industrial scale for fructooligossacharides mass-production monitoring allowing also controlling the main substrate (sucrose) and the secondary by-products (glucose and fructose). The proposed chromatographic method had a satisfactory intra- and inter-day variability (in general, with a relative standard deviation lower than 5%), high sensitivity for each sugar (usually, with a relative error lower than 5%), and low detection (lower than 0.06 ± 0.04 g/L) and quantification (lower than 0.2 ± 0.1 g/L) limits. The correct quantification of fructooligossacharides in fermentative media may allow a more precise nutritional formulation of new functional foods, since it is reported that different fructooligossacharides exhibit different biological activities and effects.

## Introduction

Fructooligosaccharides (FOS) are dietary sugars quite used as food ingredients and are usually incorporated as dietary fibers in many food products. FOS, are calorie-free and non-carcinogenic sweeteners enabling the improvement of the gastrointestinal physiology and immune functions (1–3). However, despite the current positive evidence of FOS’s effect against infections (4–6) some studies report that, under some specific conditions, FOS may cause the growth of pathogenic bacteria (7, 8). For instance, FOS reduce the colonization of *Salmonella enteritidis*, but concomitantly increase the translocation of this invasive pathogen (9, 10). On other hand, Silva et al. (11) observed that FOS reduced *Klebsiella* sp. translocation. The different behaviors observed can be a result of genetic diversity between pathogenic bacteria. Recently, Schouler et al. (12) described the presence of a gene cluster involved in the metabolism of short-chain FOS in a pathogenic *Escherichia coli* BEN2908. So, FOS’s characterization and quantification is needed and required namely for food-labeling purposes since, for instance, the use of absolute values of a specific nutrient may allow a better understanding by the consumer about possible health effects (13, 14). Different structurally oligossacharides have been referred as FOS, namely, 1-kestose, nystose, and 1^F^-fructofuranosylnystose. Accordingly, FOS are considered prebiotics with several health benefits, playing a key role in individual health and being effective against chronic inflammatory diseases (15, 16). Based on its current definition (United Nations’ Food and Agriculture Organization – FAO), a prebiotic is a non-viable food component that confers a health benefit to the host associated with modulation of the microbiota (17, 18). These compounds have a huge economic relevance in the food industry and a great health impact, being increasingly important in food and nutrition sciences (2, 3, 19). FOS are usually present in plants or fruits (15, 19–21) although in low concentrations and their individual relative proportion vary considerable from plant to plant which, from an industrial point of view, may not be economically viable to obtain them by extraction. Alternatively, FOS may be produced, either by fermentation from raw materials rich in sucrose, or by the action of enzymes with transfructosylation activity that can be derived from microorganisms (16, 22–26). Therefore, it is highly relevant to have fast, cost-effective, and accurate analytical techniques that enable the simultaneous quantification of the existent mono- and disaccharides in a fermentative medium (namely glucose, fructose, and sucrose), as well as the most common FOS (namely 1-kestose, nystose, and 1^F^-fructofuranosylnystose), which may differ in both molecular structure and weight depending on their source. Furthermore, the biological activity and its physiological effect may depend not only on the total FOS concentration but also on the specific molecular structure, although the analytical distinction and identification of a particular oligosaccharide is still difficult (27). Zdunczyk et al. (28) reported that, when compared with a kestose-rich preparation, the administration of a nystose-rich diet increased the concentration of volatile fatty acids in rats. Also, Pejin et al. (29) observed that nystose exhibit a higher anti-hydroxyl radical activity than 1-kestose, showing that the nystose can be a more active natural product. Nevertheless, FOS quantification requires the use of a systematic analytical approach, being chromatographic techniques the most widespread tools for sugar analysis (2, 19), although the majority of them are technically demanding, time-consuming, and expensive. In the last two decades, several chromatographic techniques have been proposed for FOS identification and/or quantification in plants or fruits, namely thin-layer chromatography, high-performance liquid chromatography (HPLC), and gas chromatography coupled or not to mass-spectrometry (13, 19, 22, 27, 30–33). Mass-spectrometry (MS) based-techniques are usually applied due to the low content of FOS found in plants and fruits. Nevertheless, most of these techniques present technical and analytical constrains being high-performance anion-exchange chromatography (HPAEC) with pulsed amperometric detector (PAD) and liquid chromatography MS the most used for FOS analysis (19). Borromei et al. (34, 35) applied HPAEC-PAD or HPAEC coupled with pulse electrochemical detector (PED) to quantify FOS in milks. Also, Feinberg et al. (36) proposed and validated a HPAEC-PAD method to determine complex polysaccharides, including FOS, in foods. Recently, Blanch et al. (15, 21) quantified FOS (e.g., 1-kestose, neokestose, nystose, nystose b, and kestopentaose) in food matrices using HPAEC-PAD. On the other hand, HPLC with PAD or refractive index (RI) detector have also been used to identify and quantify glucose, fructose, sucrose, and FOS derivatives kestose, nystose, and 1-fructofuranosylnystose in fermentative media (16, 23, 25, 26, 37), revealing to be a suitable routine technique considering their high levels present in fermentation samples. Other techniques have also been proposed and successfully applied for FOS detection, identification, and/or quantification, namely nuclear magnetic resonance, fluorophore-assisted carbohydrate electrophoresis, matrix-assisted desorption/ionization time-of-flight MS, or even using HPLC coupled with electrospray ionization tandem MS (19, 38, 39). Even so, these last techniques are not commonly used since they are technically demanding (19) and far beyond the economic capacity of the majority of FOS producers.

In this study, a HPLC-RI method, which uses a NH_2_ stationary phase column, is proposed and in-house validated, for the simultaneous quantification of individual FOS (1-kestose, nystose, and 1^F^-fructofuranosylnystose), mono- and disaccharides (glucose, fructose, and sucrose). The main objective was to demonstrate that a simple, fast, and cost-effective chromatographic approach could be used to accurately follow the dynamic of FOS production via sucrose fermentation using *Aspergillus aculeatus*, which could be easily implemented for screening studies at laboratorial scale or even used by industry for FOS mass-production monitoring.

## Materials and Methods

### Reagents and standards

All chemicals were of analytical grade and used as purchased. The FOS standards, 1-kestose, nystose, and 1^F^-fructofuranosylnystose (purchased from Fluka or Wako Pure Chemical Industries, Ltd., Japan) had a minimum purity of 98, 98, and 80%, respectively. Before use, all aqueous standard solutions were filtered using nylon membranes with a porosity of 0.2 μm (Puradisc 25 NYL with a diameter of 25 mm, from Whatman) and were stored at −20°C until analysis. All solvents were of analytical grade without any further purification. Acetonitrile of HPLC grade (Lab-Scan) had a minimum purity of 99.8% (supplied by Merck). Deionized water was obtained using a water purification system (TGI pure water system). Before use, all solvents were filtered using nylon membranes with a porosity of 0.2 μm (Whatman) and degassed during at least 15 min.

### HPLC analysis and equipment

A Knauer HPLC system equipped with a Knauer SmartLine 1000 pump, an automatic solvent degassing system (Knauer Manager 5000), a RI detector (RI Knauer SmartLine 2300) and a manual injector with a 20 μL loop was used. The chromatographic separation was achieved using a Knauer Eurospher 100-5 NH_2_ Vertex 25 mm × 4.6 mm column (with a security guard cartridge), at 35.0 ± 0.1°C, placed inside an oven (Grace, model 7971R). Isocratic elution was achieved using a mixture of acetonitrile and 0.04% ammonium hydroxide in water (70:30 v/v) at a flow rate of 1.25 ml/min. Each sample was analyzed in triplicate.

### HPLC-RI method in-house validation

The HPLC method used to detect and quantify mono-, disaccharides, and FOS was evaluated for linearity, detection, and quantification limits, accuracy, and precision (assays performed for repeatability and intermediate precision).

#### Intra- and inter-day HPLC injection variability

An external standard calibration methodology was applied to identify and quantify the sugars under analysis (fructose, glucose, sucrose, 1-kestose, nystose, and 1^F^-fructofuranosylnystose). Linearity was evaluated using nine sugar standard solutions with concentrations ranging from 0.2 to 25.5 g/L (Table 1). They were prepared individually by consecutive dilutions from a stock solution containing a known concentration of each compound, which was assessed by weight. Injection intra-day repeatability was studied by evaluating the relative standard deviation percentage (%RSD) of the retention times (27 injections for each sugar) and the area values (3 injections for each sugar and concentration level) from the results recorded for the triplicate analysis of each standard solution in the same day. Injection inter-day repeatability was evaluated based on the %RSD values obtained from the retention times (36 values for each sugar) and area values (4 values for each sugar and concentration level) recorded for each of the nine standard solutions analyzed once in four consecutive days.

**Table 1:** **Standard solutions concentrations used for establishing the calibration curves**.

Standard solution	Concentration, g/L					
	Fructose	Glucose	Sucrose	1-Kestose	Nystose	1^F^-Fructofuranosylnystose
P1	0.253	0.252	0.252	0.250	0.252	0.250
P2	0.506	0.503	0.504	0.500	0.504	0.500
P3	0.709	0.704	0.705	0.700	0.705	0.700
P4	1.01	1.01	1.01	1.00	1.01	1.00
P5	2.53	2.52	2.52	2.50	2.52	2.50
P6	5.06	5.03	5.04	5.00	5.04	5.00
P7	10.1	10.1	10.1	10.0	10.1	10.0
P8	20.2	20.1	20.1	20.0	20.1	20.0
P9	25.3	25.2	25.2	25.0	25.2	25.0

#### Linearity, limits of detection and of quantification

The results were plotted for evaluating the linear relationship between the peak areas of each sugar and their concentrations. Calibration curves for each sugar were established based on the data recorded during triplicate analysis carried out in the same day (intra-day variability), as well as from the chromatographic profiles recorded during four consecutive days (inter-day variability). One-way analysis of variance (ANOVA) was used to assess the statistical significance of each linear regression model being the quality of the fitted models evaluated by their correlation coefficient values (R-Pearson). The statistical significances of the slope and of the intercept values were evaluated by a *t*-test. The regression data for consecutive days were subjected to a likelihood ratio test of equality (covariance analysis) to infer about inter-day variability of the calibration curves enabling the decision if it is necessary to establish a new calibration curve whenever a new FOS quantification is required. Statistic analyses were performed using the SPSS 17 Standard Version software (SPSS INC.) at a 5% significance level. Detection (LOD) and quantification (LOQ) limits were calculated using the parameters of the calibration curves, being defined as 3.3 and 10 times the value of the regression error divided by the slope, respectively (40, 41).

#### HPLC instrumental precision, accuracy, and recovery assays

Instrumental precision was evaluated to verify the repeatability of the chromatographic analysis. The instrumental system precision was studied using three standard quality control solutions (QCS) with known concentrations (0.7, 11, and 20 g/L for each sugar) of the six sugars under study, which corresponded to low, middle, and high concentrations according to the dynamic range of the calibration curves. These levels were also evaluated since it was expected that, during the fermentation with *A. aculeatus*, FOS levels could vary from 0 to 200 g/L (37), which could be easily quantified after proper dilutions. Each solution was injected, under the working conditions, three times on the same day to evaluate the repeatability of the instrumental system (i.e., intra-day variation, considering only within day variations). The accuracy of the HPLC method was evaluated by comparing the real concentration of each sugar in each QCS and the calculated concentrations using the calibration curves previously established. The instrumental precision and accuracy were assessed by calculating the %RSD and the relative error percentage (%RE). Further, with the purpose of evaluating the recovery performance of the method, a fermentation sample was collected at 84 h of fermentation (to ensure that fructose, glucose, sucrose, and the three individual FOS were present in the sample at appreciate levels). After filtration, the sample was diluted (1:8, v/v) and split into four aliquots of 200 μL, one unfortified and the other three fortified with all the analytes at three different concentrations (25, 50, and 100 μL of a 25 g/L stock solution). This procedure was done in triplicate.

### FOS fermentation samples

The HPLC-RI method developed was applied to monitor the FOS production via sucrose fermentation by *A. aculeatus*. The fermentation inoculum (25 mL) was prepared at a spore concentration of 1 × 10^6^ spores/mL. The fermentation medium (500 mL; with 200 g/L of sucrose, 20 g/L of NaNO_3_, 0.5 g/L of KCl, 0.01 g/L of FeSO_4_.7H_2_O, 0.35 g/L of K_2_SO_4_, and 7.89 g/L of KH_2_PO_4_, pH 5.0) was prepared in a 2 L flask with baffles. After sterilization (15 min at 121°C) the fermentation was carried out in an incubator with orbital agitation (OVAN, model OPAC/ACOP) at 27°C and 150 rpm during 7 days. Twice a day a sample (5 mL) was collected and filtrated through a 0.2 μm Nylon filter (Whatman) and kept at −20°C until chromatographic analysis. Whenever necessary, samples were diluted before analysis. Three fermentation replicas were carried out.

## Results and Discussion

In this work, it was performed the in-house validation of an HPLC-RI method for the quantification of the main sugars (monosaccharides – glucose and fructose; disaccharides – sucrose; and FOS – 1-kestose, nystose, and 1^F^-fructofuranosylnystose) that may be present in the FOS production by sucrose fermentation using *A. aculeatu*s. This fungus was used since it was previously reported as one of the most promising FOS-producing fungi (42) and it has been widely described as a fructusyltransferase producer suggesting that it could be used in a single step fermentation for FOS production (3, 37).

**Figure 1:**
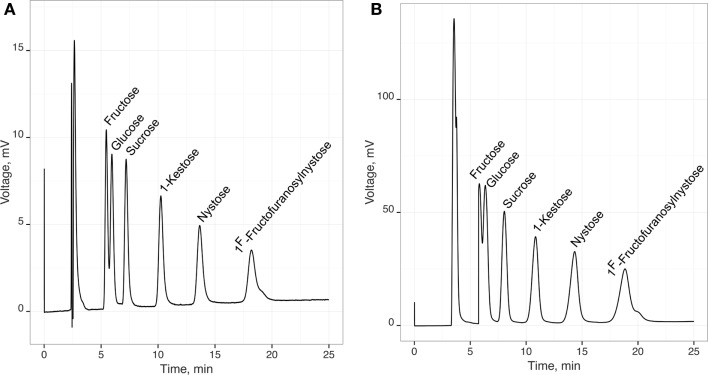
**Typical chromatogram recorded for a standard solution (A) and a fermentation sample (B) containing fructose, glucose, sucrose, 1-kestose, nystose, and 1^F^-fructofuranosylnystose**.

A typical chromatographic profile of those sugars is shown in Figure 1 that corresponds to the standard solution P4 (Table 1) containing an average concentration of each sugar of 1 g/L. In this figure, it is also shown a typical chromatogram of a fermentation sample. From Figure 1, it is clear that a good separation of the different sugars peaks can be obtained, especially regarding FOS, in a 25 min single chromatographic run, even for fermentation samples.

### Intra- and inter-day HPLC injection variability

The results obtained for intra-day and inter-day variability assays of the chromatographic injections, evaluated using the nine standard solutions, concerning retention times, and peak areas, are given in Tables 2 and 3, respectively.

**Table 2:** **Intra- and inter-day variabilities for sugars retention times (27 injections)**.

Compound	Retention time (minutes)			
	Intra-day assays		Inter-day assays	
	${\bar{t}}_{R}\pm s$	%RSD	${\bar{t}}_{R}\pm s$	%RSD
Fructose	5.57 ± 0.02	0.34	5.6 ± 0.1	2.16
Glucose	6.10 ± 0.02	0.30	6.1 ± 0.2	2.55
Sucrose	7.48 ± 0.03	0.34	7.4 ± 0.3	3.57
1-Kestose	10.84 ± 0.04	0.40	10.7 ± 0.6	5.18
Nystose	14.66 ± 0.07	0.45	14.4 ± 0.9	6.49
1^F^-Fructofuranosylnystose	19.8 ± 0.1	0.52	19 ± 1	7.74

*${\bar{t}}_{R}\pm s$: average retention time ± standard deviation; %RSD, relative standard deviation percentage*.

**Table 3:** **Intra- and inter-day repeatabilities of sugars peak areas (one injection in four consecutive days)**.

Compound	Intra-day variability: %RSD for peak areas								
	P1	P2	P3	P4	P5	P6	P7	P8	P9
Fructose	1.63	0.18	0.31	0.23	0.22	0.92	1.06	0.37	0.36
Glucose	0.74	0.20	0.26	0.38	0.27	1.19	1.14	0.31	0.40
Sucrose	3.18	1.08	0.82	0.18	0.37	1.32	1.43	0.48	0.17
1-Kestose	6.60	1.54	0.08	0.63	0.30	1.65	1.25	0.53	1.02
Nystose	5.03	0.39	1.09	0.46	0.42	1.39	1.31	0.50	0.43
1^F^-Fructofuranosylnystose	10.13	1.14	0.86	0.25	0.53	2.12	1.91	0.51	0.07

**Compound**	**Inter-day variability: %RSD for peak areas**								
	
	**P1**	**P2**	**P3**	**P4**	**P5**	**P6**	**P7**	**P8**	**P9**

Fructose	2.87	2.69	2.55	3.55	2.96	3.33	2.74	2.70	4.21
Glucose	6.95	3.96	1.37	2.94	2.32	3.43	2.53	2.76	4.12
Sucrose	3.84	3.36	1.45	2.44	0.86	2.73	0.45	1.53	1.79
1-Kestose	3.47	3.33	2.41	1.77	0.80	2.57	0.86	2.37	1.41
Nystose	3.89	3.26	3.52	1.47	0.56	2.87	0.73	1.70	1.81
1^F^-Fructofuranosylnystose	4.57	2.71	2.18	1.78	0.97	2.14	0.90	0.93	1.83

*%RSD, relative standard deviation percentage*.

Since the intra-day and inter-day %RSD values were usually lower than 5%, it is possible to conclude that the within and between days chromatographic injection variability is globally satisfactory for all the sugars analyzed (40). These results are in accordance with those reported by Borromei et al. (35) for intra- and inter-day variation of glucose, fructose, sucrose, and 1-kestose retention times in samples, analyzed using HPAEC coupled with pulse electrochemical detection (PED).

### Linearity, limits of detection and of quantification

The linearity of the HPLC-RI method, for the calculation of glucose, fructose, sucrose, 1-kestose, nystose, and 1^F^-fructofuranosylnystose, was evaluated through the calibration curves obtained by linear regression (*R* ≥ 0.9991), considering the peak area for each sugar, in arbitrary units (Figure 1), vs. the concentration of each sugar (g/L). The calibration correlation coefficients are of the same magnitude as those obtained by Borromei et al. (34) using a HPAEC coupled with PAD for similar concentration dynamic ranges.

All the linear calibration models were statistically significant, as well as all the slope values (*P* ≤ 0.001). Repeatability and intermediate precision of the calibration curves were evaluated by establishing the calibration curves based on the chromatographic runs recorded in the same day and on four consecutive days (Table 4). In both cases, two linear dynamic ranges were defined (from 0.25 to 2.5 g/L; and 2.5 and 25 g/L) allowing the determination of LOD and LOQ values (Tables 4 and 5) lower than the lowest concentration included in the experimental range (0.25 or 2.5 g/L, respectively), which were calculated from the calibration parameters (40, 41). The establishment of two linear dynamic ranges was required since a slight curvature (as can be inferred by comparing the different slope values obtained – Tables 4 and 5) was observed within the overall concentration range evaluated. Depending on the FOS concentrations found in each fermentation sample, and to avoid large dilutions, the most adequate calibration curve was used. Furthermore, a covariance analysis, applied to the calibration curves for each sugar (*data not shown*), indicated that the linear regressions obtained in four consecutive days were not statistically different (*P* ≥ 0.100), thus meaning that the same calibration curve could be used during at least 1 week for quantification purposes. Also, LOD and LOQ values obtained for intra- and inter-day analysis are, usually, of the some magnitude, showing a satisfactory precision. Moreover, from the analysis of the results shown in Tables 4 and 5, it is evident that the calculated LOD (varying from 30 to 60 μg/mL) and LOQ (varying from 100 to 200 μg/mL) values were always lower than the lowest standard concentration tested in each dynamic interval of the calibration curves, indicating a satisfactory sensitivity for each sugar standard. Overall, LOD and LOQ values are much higher than those reported [e.g., (1)] for MS based-techniques (at nanograms per milliliter level). On the other hand LOD and LOQ values of the proposed HPLC–RI method are in the same range but slightly higher than those reported in the literature for HPAEC–PAD (LOD < 0.6 μg/mL and LOQ < 2 μg/mL) (34, 36) and HPAEC–PED (LOD < 12.5 μg/mL and LOQ < 32 μg/mL) (35).

**Table 4:** **Calibration curves for the dynamic range covering concentrations between 0.25 and 2.5 g/L**.

		Compound $\left(\bar{x}\pm s\right)$					
		Fructose	Glucose	Sucrose	1-Kestose	Nystose	1^F^-Fructofuranosylnystose
Repeatability (three injections)	Slope (L/g)	128.4 ± 0.4	120.3 ± 0.4	171.9 ± 0.7	171.4 ± 0.7	155.8 ± 0.5	112.2 ± 0.2
	R-Pearson	>0.9999	>0.9999	>0.9998	>0.9999	>0.9998	>0.9997
	LOD (g/L)	0.03 ± 0.01	0.03 ± 0.01	0.04 ± 0.01	0.030 ± 0.004	0.04 ± 0.01	0.05 ± 0.03
	LOQ (g/L)	0.11 ± 0.02	0.11 ± 0.02	0.12 ± 0.03	0.10 ± 0.01	0.14 ± 0.02	0.2 ± 0.1
Intermediate precision (4 days)	Slope (L/g)	128 ± 4	120 ± 4	171 ± 2	170 ± 2	154 ± 2	110 ± 1
	R-Pearson	>0.9998	>0.9997	>0.9996	>0.9996	>0.9991	>0.9997
	LOD (g/L)	0.04 ± 0.01	0.04 ± 0.02	0.04 ± 0.03	0.04 ± 0.03	0.06 ± 0.04	0.04 ± 0.03
	LOQ (g/L)	0.12 ± 0.05	0.13 ± 0.06	0.1 ± 0.1	0.13 ± 0.09	0.2 ± 0.1	0.1 ± 0.1

*LOD, detection limit; LOQ, quantification limit; $\left(\bar{x}\pm s\right)$, average ± standard deviation*.

**Table 5:** **Calibration curves for the dynamic range covering concentrations between 2.5 and 25 g/L**.

		Compound $\left(\bar{x}\pm s\right)$					
		Fructose	Glucose	Sucrose	1-Kestose	Nystose	1^F^-Fructofuranosylnystose
Repeatability (three injections)	Slope (L/g)	132.3 ± 0.4	123.7 ± 0.4	176.0 ± 0.1	178 ± 2	159.6 ± 0.4	112.9 ± 0.1
	R-Pearson	>0.9997	>0.9997	>0.9997	>0.9996	>0.9996	>0.9994
	LOD (g/L)	0.65 ± 0.07	0.67 ± 0.08	0.6 ± 0.2	0.7 ± 0.2	0.8 ± 0.2	0.8 ± 0.2
	LOQ (g/L)	2.2 ± 0.2	2.2 ± 0.3	1.9 ± 0.7	2.4 ± 0.6	2.6 ± 0.6	2.8 ± 0.8
Intermediate precision (4 days)	Slope (L/g)	132 ± 5	124 ± 5	175 ± 3	177 ± 3	159 ± 2	112 ± 2
	R-Pearson	>0.9995	>0.9995	>0.9993	>0.9994	>0.9993	>0.9996
	LOD (g/L)	0.7 ± 0.3	0.7 ± 0.3	0.7 ± 0.4	0.8 ± 0.2	0.8 ± 0.4	0.7 ± 0.3
	LOQ (g/L)	2.2 ± 1.1	2.3 ± 1.1	2.4 ± 1.2	2.8 ± 0.8	2.8 ± 1.2	2.5 ± 0.9

*LOD, detection limit; LOQ, quantification limit; $\left(\bar{x}\pm s\right)$, average ± standard deviation*.

### HPLC instrumental precision, accuracy, and recovery assays

The instrumental precision and accuracy of the proposed HPLC–RI method were evaluated by analyzing 3 QCS with known concentrations of each sugar. The results obtained are shown in Table 6. Based on the results obtained, it can be stated that the HPLC–RI method has an acceptable precision (%RSD lower than 5%) and a global satisfactory accuracy (%RE lower, in general, than 6%) (40, 41). In Table 7, the results concerning recovery evaluation are given showing that in general, recoveries between 80 and 120% are obtained, which are in accordance with the values reported by Borromei et al. (43) regarding the application of a HPAEC–PAD method for prebiotics quantifications in fermented milk samples. Furthermore, the recoveries achieved with the present method are quite satisfactory since values between 50 and 120% are acceptable if the respective %RSD is lower than 15% (44), which is the case (%RSD < 5%).

**Table 6:** **Precision and accuracy of the HPLC–RI analysis**.

Compound	QCS1 (g/L)	Average concentration $\left(\bar{x}\pm s,\;\text{g/L}\right)$	%RSD	%RE
Fructose	0.723	0.74 ± 0.05	7.10	2.35
Glucose	0.719	0.76 ± 0.03	3.94	6.15
Sucrose	0.719	0.76 ± 0.03	4.11	6.33
1-Kestose	0.714	0.76 ± 0.04	4.94	6.08
Nystose	0.719	0.75 ± 0.04	5.95	4.80
1^F^-Fructofuranosylnystose	0.714	0.74 ± 0.01	1.96	4.26

**Compound**	**QCS2 (g/L)**	**Average concentration $\left(\bar{x}\pm s,\;\text{g/L}\right)$**	**%RSD**	**%RE**

Fructose	11.01	10.8 ± 0.4	4.01	1.79
Glucose	10.94	10.8 ± 0.4	3.75	1.42
Sucrose	10.95	10.7 ± 0.2	2.22	1.91
1-Kestose	10.87	10.6 ± 0.2	1.83	2.58
Nystose	10.95	10.8 ± 0.2	1.77	1.54
1^F^-Fructofuranosylnystose	10.87	10.6 ± 0.1	1.22	2.37

**Compound**	**QCS3 (g/L)**	**Average concentration $\left(\bar{x}\pm s,\;\text{g/L}\right)$**	**%RSD**	**%RE**

Fructose	20.26	20.0 ± 0.9	4.41	1.13
Glucose	20.13	20.0 ± 0.9	4.38	0.52
Sucrose	20.14	20.2 ± 0.3	1.73	0.05
1-Kestose	20.00	19.8 ± 0.3	1.56	0.88
Nystose	20.14	20.1 ± 0.2	1.24	0.41
1^F^-Fructofuranosylnystose	20.00	19.7 ± 0.3	1.61	1.32

*QCS, quality control solution; %RSD, relative standard deviation percentage; %RE, relative error percentage; $\left(\bar{x}\pm s\right)$, average ± standard deviation; Average concentration was calculated using the adequate linear calibration curve according to the concentration dynamic range; for QCS1 using data on Table 4; for QCS2 and QCS3 using data from Table 5*.

**Table 7:** **Recovery assays**.

Compound	Average recoveries $\left(\bar{x}\pm s,\;\%\right)$		
	25 μL (≈0.625 mg)	50 μL (≈1.25 mg)	100 μL (≈2.50 mg)
Fructose	71 ± 2	91.1 ± 0.2	94 ± 4
Glucose	92 ± 1	101 ± 1	104.0 ± 0.1
Sucrose	92.2 ± 0.1	103 ± 1	97.8 ± 0.6
1-Kestose	82.4 ± 0.9	94 ± 3	95 ± 2
Nystose	89 ± 1	98 ± 2	99 ± 2
1^F^-Fructofuranosylnystose	100.9 ± 0.2	121 ± 2	130 ± 1

*$\left(\bar{x}\pm s\right)$, average ± standard deviation*.

### Detection and quantification of FOS during fermentation process

**Figure 2:**
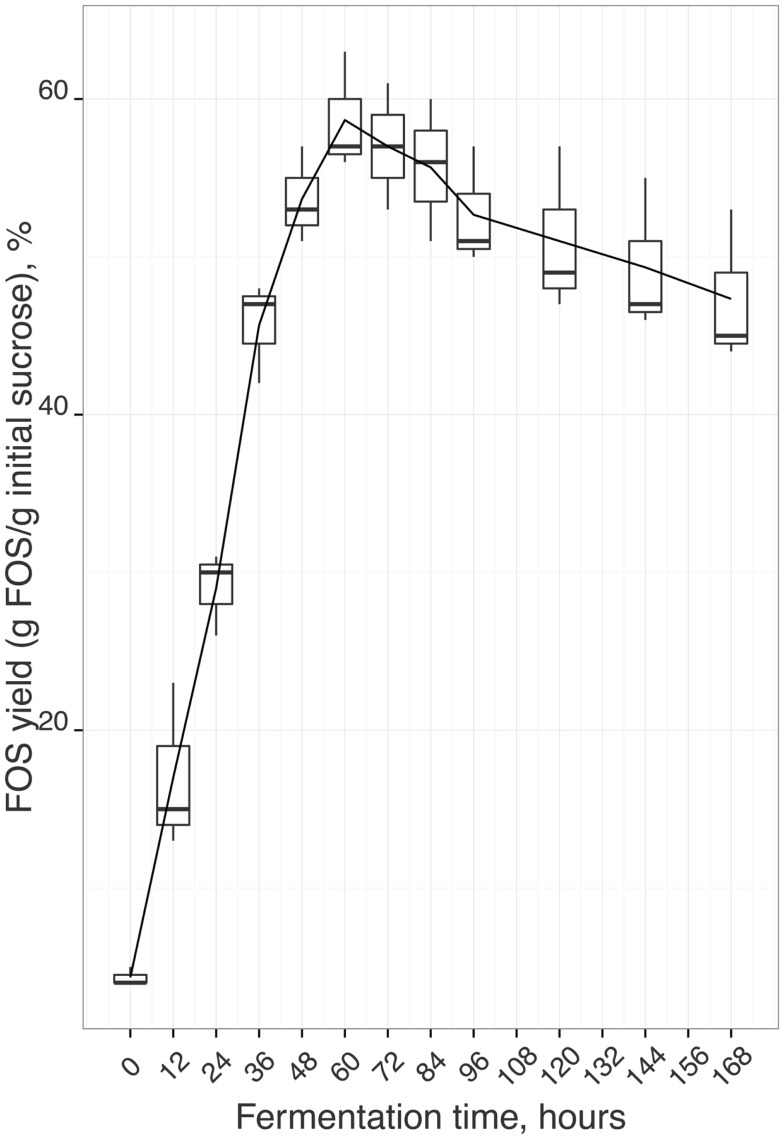
**FOS yield calculated using the in-house validated HPLC–RI method: three independent fermentations of FOS production via sucrose fermentation using *A. aculeatus* at 27°C and 150 rpm**.

**Figure 3:**
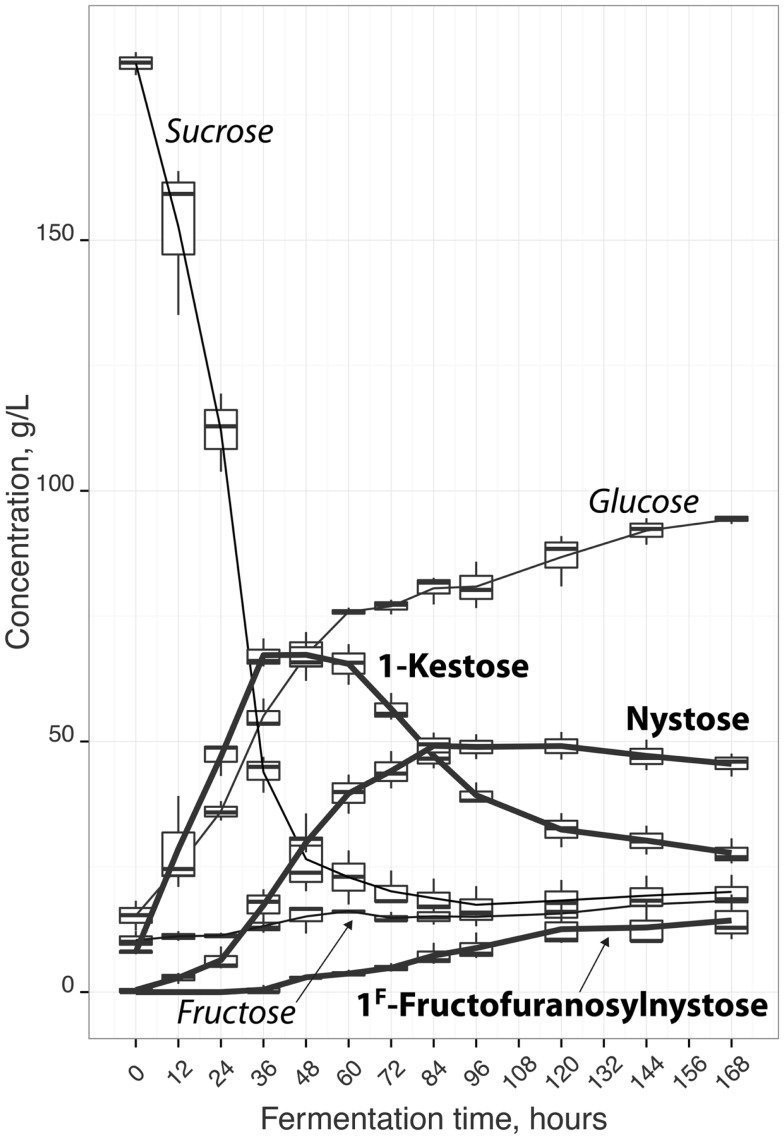
**Reproducibility (3 fermentations) of individual FOS, fructose, glucose, and sucrose levels calculated using the proposed HPLC–RI method, during sucrose fermentation (g.L^−1^) using *A. aculetus***.

Depending on the source and extraction/production conditions, different amounts and composition of an oligomer mixture can be obtained. Campbell et al. (20) observed that, in general, fruits have highest amount of 1-kestose, followed by 1^F^-fructofuranosylnystose and nystose and vegetables have highest amount of 1-kestose, followed by nystose and 1^F^-fructofuranosylnystose. However, some vegetables like chinese chive, endive, and Jerusalem artichoke have highest amounts of 1^F^-fructofuranosylnystose followed by nystose and kestose. Therefore, FOS commercial preparations from plants can have different amounts of individual FOS and/or contain also sub-products as glucose, fructose, sucrose, or others fermentable sugars, thus being difficult to establish the prebiotic features of these products. Also, FOS biological activity is related to their molecular structure (27). Indeed, some studies reported that nystose can exhibit higher biological activity than 1-kestose (28, 29). Hence, it is crucial to optimize not only the FOS yield, but also the content of each individual oligomer, in order to obtain a product with higher market value. In this context, the aim of this work was to study FOS production, via sucrose fermentation, using *A. aculeatus*, in order to obtain a product not only with a high amount of FOS but also with a known profile of individual oligomers. This imposes the use of time-consuming and expensive analytical techniques to monitor fermentation processes. Therefore, the in-house validated HPLC–RI method was applied to follow and monitor FOS production (Figure 2) during 3 independent fermentations. The methodology developed allowed quantifying 1-kestose, nystose, and 1^F^-fructofuranosylnystose and also the by-products glucose and fructose, as well as the remaining amount of sucrose (Figure 3). Figure 2 illustrates the average FOS yield profile (g FOS/g initial sucrose) along time, and reveals a satisfactory reproducibility of the fermentation process. The production of FOS was influenced by the time of fermentation and a maximum yield was obtained after 60 h of inoculation. Afterward, it was observed that the sucrose level remained almost constant, indicating that, after this moment, fungi had an apparently preference for consuming 1-kestose, thus influencing the oligomers relative ratios. As can be seen from Figure 3, when the maximum FOS yield was reached, the 1^F^-fructofuranosylnystose was present in residual amounts, and 1-kestose was the most abundant oligomer. During the following 24 h of fermentation, the total FOS amount decreases, being observed a significant change of the individual FOS profiles, with a decrease of 1-kestose content, an increase of nystose concentration reaching its maximum value, and an almost constant level of 1^F^-fructofuranosylnystose. After 84 h of fermentation, the yield of total FOS continues to decrease (Figure 2), the amount of nystose, and 1^F^-fructofuranosylnystose remains practically constant and 1-kestose continues to be degraded with the concomitantly increase of the sub-product glucose. Therefore, the fermentation period between 60 and 84 h appears to be crucial for obtaining a final product with different ratios of 1-kestose and nystose, near to their maximum levels. The in-house validated HPLC–RI method revealed to be a simple and cost-effective methodology enabling to monitor individual FOS production and their eventual consumption during a fermentation process. In fact, this analytical methodology allows the simultaneous identification of the optimal fermentation time for maximizing total or a specific individual FOS yield. So, it may allow identifying the optimal fermentation end-point, which maximizes the production of a specific oligomer, toward obtaining a final prebiotic product with a relative composition that may enhance biological activity and to improve the expected beneficial physiological effects. Finally, at industrial level, this method can be of surplus value for achieving a reproducible and homogeneous product in the minimum fermentation time. Despite the very satisfactory results obtained, as well as the potential of the proposed HPLC method for evaluating FOS production profiles during fermentation, it should be kept in mind that this methodology was specifically developed for fermentation matrices with a high content of FOS, and that the type of FOS present in these matrices were known beforehand. Thus, its application for matrices containing low amounts of FOS, such as plants and fruits extracts, may not be straightforward namely if the type of FOS in the matrices is unknown and/or no available standards exist.

## Conclusion

Fructooligosaccharides have a broad application in food, pharmaceutical, and veterinary studies, being increasingly important in food and nutrition sciences. Usually, FOS are produced by the action of enzymes possessing transfructosylating activity, which can be found both in plants and microorganisms. As such, the industrial production of FOS is highly dependent on enzymatic processes, being usually obtained through the sucrose fermentation by several fungi. Therefore, it is of major relevance to develop feasible analytical techniques that allow a fast and low-cost quantification of total and individual FOS. Their correct quantification may even allow a more precise nutritional formulation of new functional foods, since different FOS can exhibit different biological activities and effects. In this study, we demonstrated that a simple HPLC–RI method could be accurately used for the simultaneous quantification of 1-kestose, nystose, 1^F^-fructofuranosylnystose, glucose, fructose, and sucrose present in samples collected during the FOS fermentative production from sucrose using *A. aculeatus*. The chromatographic method was validated in-house, showing a satisfactory intra- and inter-day variability (in general, %RSD ≤ 5%), high sensitivity for each sugar (usually, %RE ≤ 5%), and low detection (≤0.06 ± 0.04 g/L) and quantification (≤0.2 ± 0.1 g/L) limits. Moreover, the proposed approach was fast (less than 30 min per run), cost-effective, and did not require any complex pre-treatment step. Furthermore, the proposed chromatographic based technique may be easily extended for the quantification of total or individual FOS in rich-FOS plant concentrated extracts or in food supplements, for which it is required to include FOS contents in label information. Nevertheless, the proposed method presents a limitation regarding full characterization of the fermentative product composition or its possible application to low FOS content matrices, which would require the complementary use of mass spectroscopy methods.

## Conflict of Interest Statement

The authors declare that the research was conducted in the absence of any commercial or financial relationships that could be construed as a potential conflict of interest.
